# AnnZarro: scalable, interactive exploration of cell-by-cell and gene-by-gene relationships in single-cell data

**DOI:** 10.64898/2026.10.10.758158

**Published:** 2026-10-12

**Authors:** Dominik J. Otto, Siddharth Baasri, Manu Setty

## Abstract

Cell-by-cell and gene-by-gene relationships, such as kernels and correlations, shape most single-cell analyses but are rarely inspected directly due to a lack of visualization tools that display them. AnnZarro is a point-and-click browser viewer that shows these matrices linked to an AnnData object’s cells × genes data. Using AnnZarro, experimental biologists can explore data without code and computational biologists can probe the relationships behind their analyses. One focused cell and one focused gene drive every panel. Clicking a cell paints its row of any cell-by-cell matrix over an embedding or spatial coordinates; clicking a gene paints its row of any gene-by-gene matrix over a gene plot such as a volcano. The focus also links genes to the cells where they change, and cells to the genes that distinguish them. AnnZarro reads only the matrix rows and columns each panel displays from zarr stores and hence users can explore matrices far larger than memory on a laptop or a shared server. Large datasets open on a reproducible subset, and users step through the remaining cells in parts. On a laptop, a 100,000-cell part of Tahoe-100M opens in 4 s and stays interactive. With the subset switched off, AnnZarro draws all 95.6 million cells in one plot in about 5 to 7 s. This tutorial prepares a dataset with Kompot differential abundance and expression, a diffusion kernel and gene correlations, then explores it in six worked examples. AnnZarro is available at https://annzarro.readthedocs.io .

